# ‘The WOW factors’: comparing workforce organization and well-being for doctors, nurses, midwives and paramedics in England

**DOI:** 10.1093/bmb/ldac003

**Published:** 2022-03-09

**Authors:** Cath Taylor, Karen Mattick, Daniele Carrieri, Anna Cox, Jill Maben

**Affiliations:** School of Health Sciences, University of Surrey, 30 Priestley Road, Surrey Research Park, Guildford, Surrey, GU2 7YH, UK; College of Medicine and Health, University of Exeter, Exeter, UK; College of Medicine and Health, University of Exeter, Exeter, UK; School of Health Sciences, University of Surrey, 30 Priestley Road, Surrey Research Park, Guildford, Surrey, GU2 7YH, UK; School of Health Sciences, University of Surrey, 30 Priestley Road, Surrey Research Park, Guildford, Surrey, GU2 7YH, UK

**Keywords:** healthcare professionals, workforce organization, mental health

## Abstract

**Background:**

High rates of poor mental health in healthcare staff threatens the quality and sustainability of healthcare delivery. Multi-factorial causes include the nature and structure of work. We conducted a critical review of UK NHS (England) data pertaining to: doctors, nurses, midwives and paramedics.

**Sources of data:**

Key demographic, service architecture (structural features of work) and well-being indicators were identified and reviewed by a stakeholder group. Data searching prioritized NHS whole workforce sources (focusing on hospital and community health services staff), which were rated according to strength of evidence.

**Findings:**

Key differences between professions were: (i) demographics: gender (nursing and midwifery female-dominated, doctors and paramedics more balanced); age (professions other than doctors had ageing workforces); ethnicity (greater diversity among doctors and nurses); (ii) service architecture: despite net staffing growth, turnover and retention were problematic in all professions; 41.5% doctors were consultants but smaller proportions held high grade/band roles in other professions; salaries were higher for doctors; (iii) well-being: all reported high job stress, particularly midwives and paramedics; sickness absence rates for nurses, midwives and paramedics were three times those of doctors, and presenteeism nearly double.

**Growing points:**

Sociocultural factors known to increase risk of poor mental health may explain some of the differences reported between professions. These factors and differences in service architecture are vital considerations when designing strategies to improve well-being.

**Areas timely for developing research:**

Multi-level systems approaches to well-being are required that consider intersectionality and structural differences between professions; together with inter-professional national databases to facilitate monitoring.

## Introduction

The well-being and mental health of healthcare professionals has been gaining increasing attention as a major public health concern and threat to the quality and sustainability of healthcare delivery—in the UK and globally. This has been spotlighted and further exacerbated by the COVID-19 pandemic with the added pressure on healthcare staff of delivering care in extreme circumstances.^1^

The National Health Service (NHS), one of the world’s biggest employers (and the biggest in the UK), employs nearly 1.6 million people^2^ and needs healthy, motivated staff to provide high quality patient care. However, increasing workload due to societal demand for healthcare services, combined with increasing external scrutiny of their work, has been associated with a high prevalence of mental ill-health amongst staff. Due to budget constraints and staff shortages, pressure is building in the healthcare system and this is taking its toll on staff as well as patients.^3^^,^^4^ Some commentators have described staff as the *‘shock absorbers in a system lacking [the] resources to meet rising demands’*, and suggest the current situation is not sustainable.^5^ Neglecting the well-being of healthcare staff has significant implications for staff and patients. Although the NHS as an employer has a duty of care to staff, staff well-being also affects patient care, safety and delivery. High levels of stress and burnout among NHS staff affect their ability to provide high quality care.^6–8^

In the UK, the mental health of the NHS workforce is a major issue, leading to presenteeism (working while unwell), absenteeism and loss of staff from the workforce.^4^^,^^9^ Stress among healthcare staff is greater than in the general working population and explains >25% of staff absence,^10^ and depression, anxiety, loss of idealism and empathy are also reported by nurses and doctors.^11–14^ NHS staff sickness absence rates are double the national average^15^ and are estimated to cost £1.1 billion.^4^ Multiple government and industry reports and publications have highlighted the need to reduce stress and improve mental health in NHS staff, e.g.^4^^,^^16–19^

Staff well-being is a pressing and complex problem influenced by many factors at individual, organizational, inter-professional and broader societal level. Research highlights the need for workplace policies and interventions to be informed by an in-depth understanding of such factors, and for more engagement with healthcare workers, in order to develop effective policies and interventions.^1^^,^^20^ Multiple professions and specialities are involved in the delivery of healthcare, and often share the same work environment, but they also have very different roles and responsibilities, and potentially different structural contributors to staff well-being and poor mental health.

In the NHS in England, the types of services and treatments available is determined regionally by clinical commissioning groups (CCGs). In 2020, there were 135 CCGs. NHS Trusts provide the services/treatments commissioned by the CCGs and include hospital, ambulance, mental health, social care and community services. Primary care is delivered in GP practices who work within primary care networks (PCNs). There are ~1300 PCNs currently in England, each covering a population of 30–50 000 people. The most robust and accurate workforce data available for NHS staff are the NHS Workforce statistics produced by NHS Digital (validated data extracted from the NHS Human Resource and Payroll System). Although these provide extensive data for hospital and community health service workers (covering all the types of Trust listed previously), reporting of data for primary care NHS staff is currently limited in scope.

This paper therefore focuses on hospital and community NHS staff from four professions; doctors, nurses (registered nurses only), midwives and paramedics, comparing features of these professions and how that profession’s work is structured that may be pertinent to understanding their well-being, which we have conceptualized as the ‘service architecture’. This work builds on previous work focussed on doctors^20^ Care Under Pressure (completed in 2019) and a current study focussed on nurses, midwives and paramedics: Care Under Pressure 2 (ongoing to July 2022)^1^. A key recommendation of Care Under Pressure is that policies that aim to secure the future of the NHS workforce should foster a supportive work culture in which individuals can thrive. Policies and interventions that target the individual in the absence of a supportive work culture are unlikely to succeed.^20^ As part of the ongoing work on Care Under Pressure 2 we realized the importance of investigating whether and how organizational factors—service architecture—that may differ within and between these professional groups may be important contributors to mental ill health.

We have selected these groups, because together doctors, nurses, midwives and paramedics comprise around 60% of the clinical workforce in the UK NHS. All have high rates of illness, and pressing recruitment and retention issues, but each profession also has distinct structural features. To our knowledge, this is the first time that this type of multi-professional comparative work has been undertaken. Given the evidence of poor mental health and challenges to staff well-being in the UK NHS and the current problems with recruitment and retention, it is important to gain an understanding of which contextual factors have resulted in these (unintended) impacts and to equip NHS managers, policy makers, leaders, staff, researchers and other stakeholders with this understanding. A necessary first step is to extract and collate such detail to enable comparison.

## Methods

Aim: to extract, synthesize, critically review and compare workforce demographic, service architecture and well-being data for doctors, nurses, midwives and paramedics working in hospital and community health service settings in England, in order to enhance understanding of shared and distinct contextual factors that may contribute to their poor mental health at work for the benefit of managers, policy makers, researchers, staff and other stakeholders.

### Objectives

- Identify the key workforce demographic and service architecture features that may differ within and between professional groups and be important contributors to mental ill health.
- Source and extract data regarding these workforce features and measures of well-being/mental ill-health, including assessment of the data in relation to (i) strength/accuracy of evidence; (ii) comparability across professions.
- Produce a summary of the key features and how they compare and contrast across and within the four professional groups, and describe their potential relationship to well-being/mental ill-health.

### Design

A critical review aims to go beyond description of the included sources and include a degree of analysis and conceptual innovation, resulting in a model or new interpretation of existing data.^21^

### Identification of key contextual features and stakeholder involvement

Key contextual features that may be important contributors to mental ill-health for each profession (doctors, nurses, midwives, paramedics) were brainstormed by the author team and expanded further through sharing drafts with two separate stakeholder groups formed to support wider projects on the causes of poor mental health in nurses, midwives and paramedics^2^ (Maben *et al.*, 2020b), and doctors^20^ (Care Under Pressure, and Care under Pressure 2). The stakeholders comprised doctors, nurses, midwives and paramedics—including those with self-disclosed lived experience of work-related poor mental health; representatives from relevant regulatory bodies and professional organizations; and patient/public representation. Stakeholders were asked to comment on an initial draft of the demographic, service architecture and well-being features felt to be important to capture and compare across (and within) professional groups, in particular to state if there were any omissions. Feedback suggested that our identified factors and features provided a useful summary of key statistics that could inform attempts to improve workforce well-being. Limitations in relation to lack of data specifically for the primary care workforce was noted, and we agreed that it would be beneficial to include types of settings in which different health professionals work (e.g. community, primary care, acute settings) if such data were available. Unfortunately, we have been unable to find such data in reliable sources and consistent formats, hence our decision to focus on hospital and community health service settings in England only.

### Sourcing and extracting data regarding contextual features

#### Data sources

For each key feature, searches were conducted for relevant data using a stepped approach, ordered according to the credibility and comparability of data. This began with attempts to find relevant data using NHS Digital (NHS Workforce Statistics), and/or NHS England-related sources based on the whole NHS hospital or community services workforce in England, prioritizing those sources where the data could be broken down by the four professions of interest. The most recent sources were used where possible, in order to provide the most relevant up-to-date data, but with priority given to using a slightly older source if it meant better comparability across professions. This included data from 2016 to 2021 (the majority of comparable NHS Digital data was from 2018, and NHS Staff Survey data were taken from the latest published survey results, 2020). If these searches were unsuccessful, the next step was to search profession-specific national (or UK-wide) sources such as the relevant regulatory bodies (General Medical Council for doctors; Nursing and Midwifery Council for nurses and midwives; and the Health and Care Professions Council for paramedics), or professional bodies/membership organizations (e.g. Royal Colleges for doctors, nurses and midwives and the College of Paramedics). We also asked our stakeholders to suggest data sources/contacts relevant to specific professional groups if we were struggling to access data. Following these attempts, other sources were examined such as charitable organizations/trade unions (e.g. the Kings Fund), university and other relevant websites, internet searches (e.g. via google); and searches for empirical research. For some variables the data for a profession includes other related staff, most notably for paramedics where data are often reported by NHS Digital for Ambulance Staff as a group, comprising: managers, emergency care practitioners, paramedics and ambulance technicians; and data for doctors from the NHS Staff Survey are only available for medical and dental staff combined. Moreover, NHS Digital data for Hospital and Community Health Services (HCHS) doctors include a small number of Hospital Practitioner/Clinical Assistant, who may not be medically qualified.

#### Data extraction

Data for demographic features, service architecture features and workforce well-being outcomes were extracted from the cited sources and are presented in Tables 2–4, respectively. Since data were presented in varying ways in different sources, for different professional groups and different features, it was necessary to transform some of the data to enable comparability across features and across professional groups. This was the case for any figures that had been reported as total numbers, which have been transformed into percentages (using a defined denominator) to enable comparability between staff groups.

#### Appraisal, synthesis and analysis

Data were evaluated according to the overall strength of evidence they provided ‘within’ the professional group. This was based upon an assessment of their representativeness and/or completeness in relation to the whole population of doctors, nurses, midwives or paramedics in hospital or community service settings in England; and in relation to the validity of the measure, i.e. how the data was collected (see Table 1). After appraising the data’s quality and strength ‘within’ each professional group, the data were rated in relation to the validity of comparing ‘across’ groups (Table 1). Using this approach, each row of data in Tables 2–4 has a rating (of high, moderate or low) for within group and between group comparisons. The rating tool was developed specifically for this review as there were no available tools that would allow both strength of evidence within and between professional groups. CT and AC lead appraisal process, though all ratings were reviewed and confirmed by all other authors.

**Table 1:** Rating the credibility and comparability of the evidence

	Strength of evidence ‘within’ professional group		Strength of evidence ‘between’ professional groups
Rating	Completeness/representativeness of professional group	Reliability of measure/method of data collection	Reliability of cross-comparison
Low	Data based upon a sample that is unlikely to represent the group well.	Based on subjective non-validated measure/narrative data.	Poor comparability across the groups: interpret with caution.
Moderate	Data likely to include most of professional group (or good representative sample) and/or may have other professions included with them.	Some concerns regarding validity of the measure or method of collection.	Moderate comparability: some incompatibility across groups to be taken into account.
High	Data likely to include all of the professional group.	Based on objective measure, routinely collected and high accuracy data.	Good comparability between the groups: data all from same/very similar sources.

**Table 2:** Comparison of demographic information for doctors, nurses, midwives and paramedics working in hospital and community health services

	Doctors	Nurses	Midwives	Paramedics	Source	Notes	Strength and reliability of evidence	
							Within group	Between group
Gender	^*^ 54.8% Male	^*^^*^ 11.6% Male	^*^^*^ 0.4% Male	^*^^*^^*^ 59% Male	^*^NHS digital HCHS doctors, January 2018 https://digital.nhs.uk/data-and-information/find-data-and-publications/supplementary-information/2018-supplementary-information-files/staff-numbers/consultants-and-doctors/hchs-doctors-by-specialty-grade-gender-and-age-jan-2018 ^*^^*^Nurses and midwives: https://digital.nhs.uk/data-and-information/find-data-and-publications/supplementary-information/2018-supplementary-information-files/staff-numbers/nurses-midwives-and-support-staff-by-area-level-gender-and-age-january-18 ^*^^*^^*^ HCPC. Registrant snapshot dataset (Oct 2020: paramedics) https://www.hcpc-uk.org/about-us/insights-and-data/the-register/registrant-snapshot---2020/registrant-snapshot-1-oct-20202/ Accessed 16 June 2021	^*^/^*^^*^Data for doctors and for midwives and nurses by gender were reported in numbers N and percentages % were calculated as follows: N of doctors/midwives/nurses by age × 100/N of all professionals in staff group ^*^^*^^*^ Data for paramedics by gender were reported in numbers N and percentages % were calculated as follows: N of paramedics by age × 100/N of all professionals in staff group.	High Complete samples and reliable data	Moderate Doctors/nurses/midwives all England only; paramedics UK
Ethnicity	Asian: 27.5% Chinese: 2.3% Black: 4.7% Mixed: 3.1% White:49.1% Other: 4.2%	Asian: 10.5% Chinese: 0.3% Black: 8.4% Mixed: 1.4% White: 70.6% Other: 4.3%	Asian: 2% Chinese: 0.2% Black: 6.7% Mixed: 1.7% White: 85.4% Other: 0.6%	Asian: 1.1% Chinese: 0.1% Black: 0.5% Mixed: 1.3% White: 93.9% Other: 0.3%	NHS Workforce by Gov.uk (March 2020) https://www.ethnicity-facts-figures.service.gov.uk/workforce-and-business/workforce-diversity/nhs-workforce/latest#by-ethnicity-and-type-of-role Accessed on 12 November 2020	Data reported for medical staff (junior and senior doctors, and other doctors working for hospitals and community health services), does not include GPs; nurses includes health visitors and paramedics is all ambulance staff.	Moderate Data are good representation of the professions but have others included (and some excluded)	High Same source for all groups
Nationality	^*^ UK: 70% EU: 9% Rest of the world: 16% Unknown: 5%	^*^ UK: 79% EU: 7% Rest of the world: 9% Unknown 6%	^*^^*^ UK: 87% EU: 5% Rest of the world: 2% Unknown: 6%	^*^ UK: 79% EU: 2% Rest of the world: 4% Unknown: 14%	^*^NHS Digital. Specified staff by nationality grouping as at 31 July 2018 https://digital.nhs.uk/data-and-information/find-data-and-publications/supplementary-information/2018-supplementary-information-files/staff-numbers/specified-staff-by-nationality-grouping-march-2018-july-2018 Accessed on 29 July 2021 ^*^^*^NHS Digital. Midwives by nationality grouping Sept 2018 https://digital.nhs.uk/data-and-information/find-data-and-publications/supplementary-information/2019-supplementary-information-files/staff-numbers/midwives/midwives-by-nationality-grouping---sep-2014---2018 Accessed on 22 October 2020	Data for nurses excludes health visitors All percentages calculated as follows: N of [staff group] by nationality × 100/N of all [staff group] (rounded up to nearest whole number) Data for EEA not reported as numbers very small [doctors 136; nurses 102; midwives 9; paramedics; 1)	High Complete samples and reliable data	High Same source for all groups
Age	^*^ <25: 2.6% 25–34: 33.5% 35–44: 29.3% 45–54: 22.1% 55–64: 10.7% 65+: 1.7%	^*^^*^ <25: 4.7% 25–34: 24.2% 35–44: 25.8% 45–54: 30.1% 55–64: 14.4% 65+: 0.9%	^*^^*^ <25: 5.6% 25–34: 26.3% 35–44: 24.1% 45–54: 29.1% 55–64: 14.3% 65+: 0.6%	^*^^*^^*^ <25: 5.8% 25–34: 27.2% 35–44: 25.4% 45–54:27.4% 55–64:13.1% 65+: 1.1%	^*^NHS digital HCHS doctors, January 2018 https://digital.nhs.uk/data-and-information/find-data-and-publications/supplementary-information/2018-supplementary-information-files/staff-numbers/consultants-and-doctors/hchs-doctors-by-specialty-grade-gender-and-age-jan-2018 ^*^^*^NHS digital. Nurses, midwives and support staff by area, level, gender and age, January 2018 https://digital.nhs.uk/data-and-information/find-data-and-publications/supplementary-information/2018-supplementary-information-files/staff-numbers/nurses-midwives-and-support-staff-by-area-level-gender-and-age-january-18 Accessed on 23 October 2020 ^*^^*^^*^ HCPC. Registrant snapshot dataset (October 2020: paramedics) https://www.hcpc-uk.org/about-us/insights-and-data/the-register/registrant-snapshot---2020/registrant-snapshot-1-oct-20202/ Accessed 16 June 2021	^*^/^*^^*^Data for doctors and for midwives and nurses by age was reported in numbers N and percentages % were calculated as follows: N of doctors/midwives/nurses by age × 100/N of all professionals in staff group ^*^^*^^*^Data for paramedics by age was reported in numbers N and percentages % were calculated as follows: N of paramedics by age × 100/N of all professionals in staff group. Age range was added and adjusted to those reported for nurses and midwives	High Complete samples and reliable data	Moderate Doctors/nurses/midwives all England only; paramedics UK

**Table 3:** Comparison of ‘service architecture’ information for doctors, nurses, midwives and paramedics working in hospital and community health services

	Doctors	Nurses	Midwives	Paramedics	Source	Notes	Strength and reliability of evidence	
							Within group	Between group
Size of workforce	2018: 109 109 FTE *(116 423 headcount)* 2019: 111 950 FTE *(119 530 head count)* 2020: 121 256 FTE *(128 962 headcount)*	2018: 284 073 FTE *(318 595 headcount)* 2019: 288 851 FTE *(324 062 headcount)* 2020: 302 293 FTE *(338 256 headcount)*	2018: 21 601 FTE *(26 130 headcount)* 2019: 21 670 FTE *(26 245 headcount)* 2020: 22 136 FTE *(26 778 headcount)*	2018: 20 646 FTE *(21 900 headcount)* 2019: 5776 FTE *(16 775 headcount)* 2020: 16 940 FTE *(18 000 headcount)*	2018 (May) data taken from: https://digital.nhs.uk/data-and-information/publications/statistical/nhs-workforce-statistics/may-2018 2019 (May) data taken from: https://digital.nhs.uk/data-and-information/publications/statistical/nhs-workforce-statistics/may-2019 2020 (May) data taken from: https://digital.nhs.uk/data-and-information/publications/statistical/nhs-workforce-statistics/may-2020	Numbers of NHS Hospital and Community Health Service (HCHS) staff working in NHS Trusts and CCGs in England (excluding primary care staff).	Moderate Generalized evidence for nurses (including health visitors) and for paramedics (data are for ambulance staff)	High Data for all professions from the same source
Type/band **(**agenda for change)	^*^ Doctors in a formalized training post: 49.3% Doctors in a non-consultant and non-formalized training role: 8.7% Consultant: 41.5%	^*^^*^ Band 5: 42% Band 6: 28% Band 7: 15% Band 8a: 3% Band 8b: <1% Band 8c: <1% Band 8d: <1% Band 9: <1%	^*^^*^ Band 5: 8% Band 6: 55% Band 7: 15% Band 8a: 1% Band 8b: <1% Band 8c: <1% Band 8d: 0 Band 9: 0	^*^^*^ Band 5: 26% Band 6: 80% Band 7: 7% Band 8a: 1% Band 8b: <1% Band 8c: <1% Band 8d: <1% Band 9: <1%	^*^ https://files.digital.nhs.uk/9B/7D0567/NHS%20Workforce%20Statistics%2C%20May%202020%20Doctors%20by%20Grade%20and%20Specialty.xlsx Data for May 2020 ^*^^*^https://digital.nhs.uk/data-and-information/find-data-and-publications/supplementary-information/2018-supplementary-information-files/staff-numbers/hchs-staff-excluding-doctors-by-afc--staff-group Total numbers employed (to work out percentages) were taken from: Midwives: https://digital.nhs.uk/data-and-information/find-data-and-publications/supplementary-information/2018-supplementary-information-files/staff-numbers/registered-midwives-by-5-year-age-band-in-nhs-trusts-and-ccgs-in-england---september-2012-2017-november-2017 Nurses (inc health visitors): https://digital.nhs.uk/data-and-information/find-data-and-publications/supplementary-information/2018-supplementary-information-files/staff-numbers/nurses-and-health-visitors/nurse-and-health-visitor-numbers-september-2012-to-2017 Paramedics (ambulance staff) https://digital.nhs.uk/data-and-information/find-data-and-publications/supplementary-information/2018-supplementary-information-files/staff-numbers/paramedics-in-nhs-ambulance-trusts-in-england-september-2017-supplementary-information Total staff are headcount (not FTE)		High Complete samples and reliable data	Moderate Band comparisons are possible for nurses, midwives and paramedics but not for doctors. Sources use different years
Turnover	Change: +5.4% Joiners: 23 629 (UK 59.4% EU/EEA 8.8% RoW 29.7%) Leavers: 17 612 Workforce: 112 143^*^	Change: +2.8% Joiners: 39 918 (UK 68.4% EU/EEA 5.9% RoW 24.1%) Leavers: 31 829 Workforce: 289 759^*^^*^	Change: +2.2% Joiners: 3168 (UK 91.5% EU/EEA 5.4% RoW 1.2%) Leavers: 2677 Workforce: 21 873	Change: +0.5% Joiners: 1715 (UK 74.2% EU/EEA 3.5% RoW 15.9%) Leavers: 1599 Workforce: 21 380^*^^*^^*^	NHS digital. Turnover by staff group and nationality February 2019–February 2020. https://digital.nhs.uk/data-and-information/supplementary-information/2020/turnover-by-staff-group-and-nationality-feb19-and-feb20-mb21052020 Accessed on 12 November 2020 NHS Hospital & Community Health Service monthly workforce statistics. Sep 2009–May 2020 https://files.digital.nhs.uk/B2/B56CB3/NHS%20Workforce%20Statistics%2C%20May%202020%20Staff%20Group%2C%20Care%20Setting%20and%20Level.xlsx Accessed on 31 March 2021 NHS Workforce Statistics, July 2019 Doctors by Grade and Speciality https://files.digital.nhs.uk/15/58B272/NHS%20Workforce%20Statistics%2C%20July%202019%20Doctors%20by%20Grade%20and%20Specialty.xlsx Accessed on 31 March 2021	Workforce numbers by profession reported for Feb 2019 Leaver/joiner data reported for February 2019 to February 2020 Change indicates leavers subtracted from joiners and is calculated as a percentage of the workforce	Moderate ^*^ Generalized evidence for doctors (including dentists) ^*^^*^Generalized evidence for nurses (including health visitors) ^*^^*^^*^Generalized evidence for ambulance staff (including managers)	High Data for all professions from the same source
Retention (I often think about leaving this organisation)	21.4%	27.5%	35.9%	40.6%	NHS Staff Survey Results 2020 https://www.nhsstaffsurveys.com/results/interactive-results/ Accessed on 19 August 2021	The % of staff responding ‘agree’ or ‘strongly agree’ to the statement ‘I often think about leaving this organisation.’	Moderate Reliable data representing respondents to staff survey. Generalized evidence for doctors (including dentists).	High Data for all professions from the same source
Undergraduate Student attrition (drop-out rates)	^*^ 5%	^*^^*^ 24%	^*^^*^ 21%	Not available	^*^NHS Reality. An NHS soapbox. Speakers' corner for the NHS. https://nhsreality.wordpress.com/2017/09/03/300-med-student-dropouts-out-of-6000-5/ Accessed on 23 October 2020 ^*^^*^A critical moment: NHS staffing trends, retention and attrition https://www.health.org.uk/sites/default/files/upload/publications/2019/A%20Critical%20Moment_1.pdf	Data on drop-out rates from education	Moderate	Moderate Attrition rates are from a different source for doctors and not available for paramedics
Retirement age	^*^ June 2017–June 2018 Data HCHS Doctors: 15 095 (all reasons for leaving) Retirement age: *n* = 761 (5%) Average age 61.1 Voluntary early retirement: 91 (0.6%)^ Average age 57.6 Flexi-retirement n = 31 (0.2%) Average age 56.3	^*^ June 2017–June 2018 Data Nurses (incl. Health visitors): 26 776 (all reasons for leaving) Retirement age: 4466 (16.7%) Average age 58.4 Voluntary early retirement: 620 (2.3%)^ Average age 55.6 Flexi-retirement n = 185 (0.7%) Average age 56.8	^*^^*^ October 2016 to October 2017 Data Midwives: 2620 (all reasons for leaving) Retirement age: 428 (16.3%) ^*^^*^^ Average age 58.1	Not available	^*^NHS Digital: Leavers from the NHS by age and reason for leaving 2010–2019 AH3120. https://digital.nhs.uk/data-and-information/find-data-and-publications/supplementary-information/2019-supplementary-information-files/leavers-and-joiners/leavers-from-the-nhs-by-age-and-reason-for-leaving Accessed on 08 December 2020 ^*^^*^NHS Digital: Leavers from the NHS that were midwives by age with reason for leaving of retirement age. https://digital.nhs.uk/data-and-information/find-data-and-publications/supplementary-information/2018-supplementary-information-files/leavers-and-joiners/leavers-from-the-nhs-that-were-midwives-by-age-with-reason-for-leaving-of-retirement-age Accessed on 08 December 2020	^Data for leavers from the NHS by reason for leaving was reported in numbers N and percentages % were calculated as follows: N of leavers by reason of leaving × 100/N of leavers by all reasons of leaving ^*^Data reported for January 2017 to January 2018 ^*^^*^Data reported for October 2016 to October 2017	Moderate ^*^Generalized evidence for nurses (including health visitors)	Moderate Sources are comparable for doctors, nurses and midwives but data not available for paramedics.
Bank Staff Headcount percentage of total staff in 2018 England	10.5% (12 252/116 191)	18.9% (60 004/317 884)	13.8% (3598/26 062)	4.4% (969/21 934)	NHS digital. Bank staff numbers June 2018. https://digital.nhs.uk/data-and-information/find-data-and-publications/supplementary-information/2018-supplementary-information-files/bank-staff/bank-staff-numbers-june-2018 Accessed on 11 November 2020 NHS workforce statistics- June 2018 https://files.digital.nhs.uk/25/8BF819/NHS%20Workforce%20Statistics%2C%20July%202018%20National%20and%20HEE.xlsx	There were 181 073 bank staff in total across NHS trusts & CCGs in England in 2018, broken down as follows: HCHS doctors—12 252 Nurses and HVs—60 004 Midwives—3598 Ambulance staff—969 Figures represent payments made using Electronic Staff Record (ESR) system to NHS staff who are employed and directly paid by NHS organisations. Unlikely to represent all bank staff. Across NHS Trusts and CCGs in England in June 2018: HCHS doctors—116 191 nurses and HVs—317 884 midwives—26 062 Ambulance staff—21 934 Figures represent headcount totals not fulltime equivalents 2018	Moderate Data are reported in groups beyond those of interest (e.g. nurses include health visitors, and paramedics is encompassed within all ambulance staff). Health visitors only represent about 3% of the population.	High Data for all professions are from the same source
Staff vacancies	^*^ 6.1% vacancy rate (Includes doctor and dentists)	^*^ 10.3% vacancy rate (includes nurses, midwives and health visitors)	^*^ 10.3% vacancy rate (includes nurses, midwives and health visitors)	^*^^*^ 12% vacancy rate	^*^NHS Vacancy Statistics England April 2015 – June 2020 https://digital.nhs.uk/data-and-information/publications/statistical/nhs-vacancies-survey/april-2015---june-2020-experimental-statistics Accessed on 19 August 2021 ^*^^*^Recruitment and retention of ambulance staff. https://www.unison.org.uk/content/uploads/2015/11/Recruitment-and-retention-of-ambulance-staff-PRB-November-2015-FINAL.pdf Accessed on 26 October 2020	^*^Vacancies by profession reported for June 2020 The vacancy rate is a calculation of the FTE number of vacancies as a percentage of planned FTE workforce levels. Data from NHS England and NHS Improvement	Moderate ^*^Data are reported in groups beyond those of interest Doctors include dentists and nurses include midwives and health visitors)	Moderate Doctors/Nurses/Midwives all England only; Paramedics UK
Average annual basic pay (per FTE)	£68 777	£34 275	£36 059	£33 487	Annual basic pay data taken from: https://digital.nhs.uk/data-and-information/publications/statistical/nhs-staff-earnings-estimates/march-2021		Moderate Data only include NHS earnings	High Data for all professions from the same source
Pay gap (by gender)Pay gap (by ethnicity)	M: £5841 F: £5082 Difference: M 15% > F WHITE: £5734 BAME: £5153 Difference: White 10% > BAME	M: £2730 F: £2699 Difference: M 1% > F WHITE: £2762 BAME: £2568 Difference: White 7% > BAME	M: £2936 F: £2826 Difference: M 4% > F WHITE: £2820 BAME: £2851 Difference: White 1% > BAME	M: £2415 F: £2292 Difference: M 5% > F WHITE: £2368 BAME: £2258 Difference: White 5% > BAME	NHS Digital. Ethnicity pay gap FTE basic pay comparison tool—by staff group https://digital.nhs.uk/data-and-information/publications/statistical/nhs-workforce-statistics/nhs-workforce-statistics---march-2019-provisional-statistics Accessed on 31 March 2021	Using this tool and selecting the staff group of interest on the base sheet we have then selected the variables of interest on the right side of the spreadsheet (e.g. ethnic group or gender) For ethnicity, BAME represents the average pay across the following ethnic groups: - Asian/Asian British - Black/African/Caribbean/Black British - Mixed/Multiple ethnic groups - Other ethnic group - The only group excluded is ‘unknown’	Moderate Generalized evidence for nurses (including health visitors) and for paramedics (data are for ambulance staff)	High Data for all professions from the same source
Shift work patterns	Doctors may work up to 48 hours a week (but many opt-out of EWTD and work in excess^*^).	Nurses usually work standard hours of 37.5 hours per week. Many nurses will work 8, 10 or 12-hour shifts across the 24 hour day, especially in hospital settings. Some work the traditional Monday–Friday 9–5 pm shifts with weekends off or various times throughout the week while rotating the weekends.	Midwives usually work 37.5 hours per week. Midwives working on maternity wards are likely to work 12 hour shifts, while those in the community are more likely to work a 9–5 day but could be on call for home births	Paramedics usually work 37.5 hours per week on a shift pattern of 6, 8, 10 or 12-hour shifts.	NHS Health Careers. www.healthcareers.nhs.uk Compare roles: Add GP, adult nurse, midwife, paramedic Accessed 16 June 2021 ^*^Junior doctors who opt out of the European Working Time Directive (EWTD) https://www.bma.org.uk/pay-and-contracts/working-hours/european-working-time-directive-ewtd/juniors-who-opt-out-of-the-european-working-time-directiveb Accessed 8 February 2022		Moderate Generalized evidence for doctors (based on GP) and nurses (based on adult nurse).	High Data for all professions from the same source
%Staff working additional unpaid hours	75.3% (31 212/ 41 403)	64.3% (91 165/ 141 788)	43.4% (4295/ 9897)	79% (13 837/ 10 932)	NHS Staff Survey National Interactive Tables (2020) https://public.tableau.com/app/profile/piescc/viz/ST20nationaldashboards_16215084823020/Aboutthesurvey Accessed on 8 February 2022		Moderate Representative samples but relies on self-report	High Data for all professions from the same source
Education/ clinical practice	5 years undergrad 2 years foundation training Registration GMC upon completion of Y1 Foundation Programme 3–7 years. Specialization^*^ Clinical practice**^** 2% in Year 1 4% in Year 2 28% in Year 3 27% in Year 4 61% in Year 5	3 years undergrad Registration with NMC^*^^*^ Clinical practice^*^^*^ 50% practice-based learning (2300 h)	3 years undergraduate education Registration with NMC^*^^*^ Clinical practice^*^^*^ 50% practice-based learning (2300 h)	2–4 years approved qualification in paramedic science Registration HCPC^*^^*^^*^ Clinical practice**^^** 50% practice-based learning	^*^GMC. https://www.gmc-uk.org/education/becoming-a-doctor-in-the-uk Accessed on 23 October 2020 ^*^^*^NMC. https://www.nmc.org.uk/education/becoming-a-nurse-midwife-nursing-associate/ Accessed on 23 October 2020 ^*^^*^^*^College of Paramedics https://www.collegeofparamedics.co.uk/COP/Become_a_Paramedic/COP/BecomeAParamedic/Become_a_Paramedic.aspx?hkey=f10838de-b67f-44a0-83b7-8140d8cdba83 Accessed on 23 October 2020 ^University of Exeter website ^^University of Surrey website	To become a registered doctor a minimum of 6 years To become a registered nurse or midwife a minimum of 3 years. To become a registered paramedic a minimum of 2–4 years	Moderate Reliable evidence regarding curriculum via GMC and NMC and HCPC. Clinical Practice Hours variable for Doctors and Paramedics and sources are therefore examples.	Moderate Clinical Practice Hours are not taken from similar sources for Doctors and Paramedics.
CPD	50 hours of CPD per year Recommended 250 credits over 5 years^*^	35 hours over 3 years 20 of which participatory learning (activity with other professionals e.g. conference, training) ^*^^*^	35 hours over 3 years 20 of which participatory^*^^*^	No set number of hours^*^^*^^*^	^*^Royal College of Physicians: https://www.rcplondon.ac.uk/education-practice/advice/cpd-revalidation Accessed 04 November 2020 ^*^^*^NMC. http://revalidation.nmc.org.uk/what-you-need-to-do/continuing-professional-development.html Accessed 04 November 2020 ^*^^*^^*^HCPC Continuing professional development and your registration. Information for registrants https://www.hcpc-uk.org/globalassets/resources/guidance/continuing-professional-development-and-your-registration.pdf?v=637106442760000000 Assessed 04 November 2020		High Sources are reliable for the individual professions	High Sources are comparable – Regulatory/ professional bodies.

**Table 4:** Comparison of ‘workforce wellbeing’ information for doctors, nurses, midwives and paramedics working in hospital and community health services

	Doctors	Nurses	Midwives	Paramedics	Source	Notes	Strength and reliability of evidence	
							Within group	Between group
Sickness absence/ sick leave rates	1.49%^*^	4.73%	5.11%	5.38%	NHS Digital. NHS Sickness Absence Rates, April 2019 to March 2020, Annual Tables https://digital.nhs.uk/data-and-information/publications/statistical/nhs-sickness-absence-rates/march-2020 Accessed on 21 October 2020	Data reported for April 2019–March 2020 ^*^Data reported for Hospital and Community Health Service (HCHS) Doctors	Moderate Generalized evidence for nurses (including health visitors); and paramedics (ambulance staff)	High Data for all professions from the same source
% Sickness absence due to anxiety; stress; depression; other psychiatric illness	24.1%^*^	30.2%	34.7%	25.5%	NHS digital. Sickness Absence by Reason and Staff Group, June 2020. https://digital.nhs.uk/data-and-information/publications/statistical/nhs-sickness-absence-rates/june-2020 Accessed on 10 November 2020	There is also data available on reasons not related to mental health problems Data reported for June 2020 ^*^Data reported for HCHS Doctors	Moderate Generalized evidence for nurses (including health visitors); and paramedics (ambulance staff)	High Data for all professions from the same source
Presenteeism (working when unwell)	30.3%	49.3%	55.3%	56.3%	NHS Staff Survey Results 2020 https://www.nhsstaffsurveys.com/results/interactive-results/ Accessed on 19 August 2021	% staff responding ‘yes’ to the question ‘In the last three months have you ever come to work despite not feeling well enough to perform your duties?’	Moderate Reliable data representing respondents to staff survey. Generalized evidence for doctors (including dentists).	High Data for all professions from the same source
Unrealistic time pressures	80.8%	81.5%	89.7%	81.9%	NHS Staff Survey Results 2020 https://www.nhsstaffsurveys.com/results/interactive-results/ Accessed on 19 August 2021	% staff responding ‘sometimes’, ‘often’ or ‘always’ to the question ‘I have unrealistic time pressures’	Moderate Reliable data representing respondents to staff survey. Generalized evidence for doctors (including dentists).	High Data for all professions from the same source
Stress (report feeling unwell as a result of work-related stress)	39.8%	48.5%	54.9%	58.2%	NHS Staff Survey Results 2020 https://www.nhsstaffsurveys.com/results/interactive-results/ Accessed on 19 August 2021	The % of staff answering ‘yes’ to the question ‘During the last 12 months have you felt unwell as a result of work-related stress?’	Moderate Reliable data representing respondents to staff survey. Generalized evidence for doctors (including dentists).	High Data for all professions from the same source
Suicide	2015: 14 2016: 15 2017: 19 2018: 18^*^	2015: 43 2016: 51 2017: 32 2018: 55	2015: 2 2016: 1 2017: 1 2018: 3	2015: 4 2016: 5 2017: 5 2018: 3	Office for National Statistics. Number of suicides among health professionals, England and Wales, 2011–2018 https://www.ons.gov.uk/peoplepopulationandcommunity/birthsdeathsandmarriages/deaths/adhocs/10471numberofsuicidesamonghealthprofessionalsenglandandwales2011to2018 Accessed on 23 October 2020	Data reported for 2015 to 2018 ^*^Data for doctors is ‘Medical Practitioners’ Data reported as cases per year N not % Data include England and Wales	Moderate Cause reported as intentional harm or injury/poisoning of undetermined intent: may not be reliable.	High Data for all professions from the same source

## Results

Tables 2–4 provide comparative data for four key professions within the NHS hospital and community services workforce in England. The tables facilitate comparison across the different professional groups and draw attention to the key features of the professional contexts that may contribute to well-being or mental ill-health of these critical NHS staff. In the narrative summary below, we present the information relating to three categories: Demographics, Service Architecture and Workforce Well-being.

### Demographics

The professions of nursing and midwifery are heavily female dominated, with only 11.6% and 0.4% male staff, respectively (Table 2). The professions of medicine and paramedic science are more gender balanced with 54.8% and 59% male staff, respectively. In terms of ethnicity, there are striking differences. Very high proportions of midwives and paramedics (85.4% and 93.9%) report their ethnicity as White, compared to 49.1% of doctors and 70.6% of nurses. Over a quarter (27.5%) of doctors report their ethnicity as Asian, compared to 10.5% of nurses and 1–2% of midwives and paramedics. There also appear to be more nurses identifying as Black (8.4%) and more doctors identifying as Chinese (2.3%) than other professions. At least 70% of all four professions report their nationality as UK. The medical profession has the most members from the EU (9%) and from the rest of the world (16%) followed by nurses (7% EU and 9% rest of the world). In terms of age, there are quite different pictures, with the highest proportion of doctors (33.5%) in the age 25–34 category, whereas for nurses and midwives the highest proportions (30.1%, 29.1%) were in the age 45–54 category. Paramedics had similar proportions in each of these age categories (27.2% aged 25–34; 27.4% aged 45–54).

### Service architecture

Service architecture is our way of conceptualizing the structural features of a profession, including a focus on features that may be pertinent to understanding their well-being (Table 3).

### Size and types of workforce

In terms of size, nursing is by far the biggest profession, with 302 293 full-time equivalent (FTE) qualified nurses in the NHS hospital and community services workforce in 2020. Medicine is second largest with 121 256 FTE qualified doctors, followed by 22 136 FTE midwives and 16 940 FTE ambulance staff (of which the majority are paramedics). When we look at the ‘type’ of qualified staff, it is notable that 41.5% doctors are in the highest-grade category (consultant), whereas there are very few nurses, midwives or paramedics in the higher banded roles. The majority of nurses are in the lowest band (42% band 5); whereas midwives and paramedics are typically initially appointed into band 6 roles and the majority of the workforce are employed at this level (55% midwives; 80% paramedics). This suggests very different career trajectories for doctors, nurses, midwives and paramedics.

### Staff turnover, retention and retirement

Data suggest a positive trajectory in the size of the NHS workforce. Between February 2019 and February 2020, there was a net growth in number of doctors (+5.4%), nurses (+2.8%), midwives (+2.2%) and ambulance staff (+0.5%). Of those joining the NHS, 59.4% doctors, 68.4% nurses, 91.5% midwives and 74.2% paramedics were from the UK, with the remainder from the EU/EEA and the rest of the world. In the latest NHS Staff Survey (2020), over a third of paramedics (40.6%) and midwives (35.9%) reported often thinking about leaving the organization they worked in, compared to 27.5% of nurses and 21.4% doctors. Similarly nursing and midwifery students were more likely to drop out of their undergraduate courses than doctors (24% and 21% compared to 5% medical students). It is also important for workforce planning to consider the age at which healthcare staff retire. The average retirement age was similar for doctors, nurses and midwives (61.1 years; 58.4 years, 58.1 years, respectively) yet only 21% of paramedic retirements in 2018/19 were aged 60+, compared to 36% for all NHS workers.^22^

### Bank staff and vacancies

Nurses have the highest proportion of bank staff, at 18.9%, compared with 10.5% doctors (where they are more commonly called locums), 13.8% midwives and 4.4% ambulance staff. The data relating to staff vacancies are less readily comparable, but show that in England there is a 6.1% vacancy rate in doctors and dentists; 10.3% in nurses, midwives and health visitors taken together; and in the UK there is a 12% vacancy rate in paramedics.

### Salary and pay gaps

The average annual basic pay for doctors (£68 777) is nearly double that of the other three professions, with midwives (£36 059) earning slightly more than nurses (£34 275) and paramedics (£33 487). It is important to note that this figure only includes NHS earnings, and excludes any additional salary from private practice. Doctors are also more likely to receive additional payments for working on-call (34.3% vs 17.4% midwives, 7.9% ambulance staff and 4.1% nurses, data taken from same source as salary). Across all four professions, there is a gender pay gap with average pay for female staff less than the average pay for male staff, and this varies from 1% in nursing to 15% in medicine. Across all four professions, the average pay for staff who report as BAME (Black, Asian or Minority Ethnic) in terms of ethnicity is less than the average pay for staff who report as White, and this varies from 1% in midwifery to 10% in medicine.

### Working hours

All four professions typically work shifts or extended days, often involving early mornings, evenings, nights and weekends. A full-time doctor may be contracted to work up to 48 hour per week, in a range of shift patterns, dependent on specialty. Full time nurses and midwives are typically contracted to work 37.5 hour per week, through 8, 10 or 12 hour shifts in hospital settings, although working hours are often more traditional in community settings, albeit sometimes with on call commitments. Paramedics are typically contracted to work 37.5 hour per week on a shift pattern of 6, 8, 10 or 12 hours. Results from the latest NHS staff survey (2020) show that a significant proportion of staff work unpaid hours in addition to these contracted hours. Over three quarters of paramedics (79%) and doctors (75.3%) reported working additional unpaid hours, compared to 64.3% nurses and 43.4% of midwives.

### Education and training

All four professions now require a university degree for entry to the profession and all four are required to pass examinations to allow them to register as a professional with their respective registering body. As undergraduates, doctors spend a smaller proportion of their time in clinical practice (around 25% overall), whereas the other three professions spend 50% of their time as undergraduates on placements in clinical practice. Doctors also spend much longer in training, both as undergraduates and after graduation, compared to the other three professions. Medical training typically involves 5 years of undergraduate study and 5–9 years of postgraduate training, whereas the other three professions typically involve 3 years of undergraduate study and have no requirement for postgraduate training (though many opportunities exist, including advanced practice Masters and doctoral qualifications and specialist practitioner courses).

### Continued professional development

There are fixed requirements for continued professional development (CPD) in terms of numbers of hours per year for doctors, nurses and midwives, but no set number of hours for paramedics. For example, doctors who are members of the Royal College of Physicians are expected to undertake 50 hours of CPD per year, whereas for most nurses and midwives it is 35 hours over 3 years (~12 hour per year).

### Workforce well-being

Sickness absence appears to be significantly higher in nurses, midwives and paramedics (4.73%, 5.11% and 5.38%) compared to doctors (1.49%; Table 4). When we look at the proportion of sickness absence due to anxiety/stress/depression/other psychiatric illness, this ranges from 24.1% for doctors to 34.7% for midwives. Presenteeism also appears to be higher in nurses, midwives and paramedics (49.3%, 55.3% and 56.3%, respectively reporting working when unwell in the NHS Staff Survey 2020) compared to 30.3% of doctors. The majority of all four professions report having unrealistic time pressures (between 80.8% of doctors to 89.7% midwives), and high proportions in each profession reported feeling unwell as a result of work-related stress (from 39.8% medical and dental staff to 58.2% paramedics). Data were also extracted for 2018 and 2019 in case there was a ‘pandemic’ effect of using the 2020 NHS staff survey data, but we found no evidence of this with little change in these variables in any of the groups over this period.

## Discussion

The mental health and well-being of healthcare workers has been a pressing concern for many years, and has been intensified by the ongoing COVID pandemic.^1^^,^^23^ Poor mental health is the consequence of a complex interplay of bio-psycho-social-cultural factors, among these, the nature and structures of healthcare work may be major contributors. Although some of the features of work relating to poor mental health are common to all NHS staff, some key features and patterns indicate unique differences that are important to note and take into account when designing, implementing and evaluating interventions to improve well-being of NHS staff. This review presents some of this data, providing a resource to support this endeavour.

In relation to demographics, there are some stark differences by gender and whilst our focus is on work factors in this paper, various social and economic factors can put women at greater risk of poor mental health than men and thereby may go some way to explaining the high prevalence of poor mental health in the female dominated professions. These factors include being more likely to undertake caring roles, live in poverty and experience domestic abuse.^24^ Furthermore, female dominated professions may be more open to reporting poor mental health. In relation to age profile of the workforce, medicine has a younger workforce, and nursing and midwifery have an ageing workforce. This suggests that there may be greater problems with workforce retention in medicine and/or that the peak at an earlier age in medicine is the result of greater investment in medical student numbers working their way through the system. This also indicates that there are difficult times ahead for nursing and midwifery, as many experienced professionals near retirement. In nursing this has been referred to as a demographic timebomb.^25^ It is critically important to consider ways of encouraging the next generation into healthcare careers. We know that career choices for Generation Z (those born 1995–2010, so those entering the labour market now) are influenced by wanting to work for organizations that promote healthy practices and healthy working environments,^26^ and research has shown the potential ‘fit’ between Generation Z values and caregiving careers.^27^

In relation to diversity, the professions with lower ethnic diversity (nursing, midwifery and paramedic science) also have the highest vacancy rate. There are also considerable gender and BAME pay gaps across professions. In medicine the gender pay gap has been explored more comprehensively than the data we used here allows, and a greater gap than reported here was found (18.9% for hospital and community health services doctors, 15.3% for GPs, adjusted for differences in working hours).^28^ The Workforce Race Equality Standard^29^ highlights variations in staff experience according to ethnicity, across NHS trusts in England, and is challenging race inequality in the health and care system. Policies and strategies that aim to improve equality, diversity and inclusion within and across professions are not only a moral imperative, but are likely to improve recruitment and retention in Generation Z cohorts, improve the well-being of staff (e.g. reducing potential stigma and unprofessional behaviours including bullying) and also improve quality of patient care.^30^

In relation to ‘service architecture’ the four professions have many distinct features that may be important when trying to understand the causes of poor mental health. Although there has been net growth in numbers within each profession, there has also been an exponential growth in demand, and this is within a context of chronic under-investment and staff shortages,^31^ and an exacerbation of the shortages caused by Brexit.^32^ Thus it is unlikely that this growth in numbers will be sufficient. Furthermore, the numerical staffing levels we have reported can mask nuances that are important to consider, for example which NHS staff (in terms of grade and experience) are leaving and joining and the employment status of staff (e.g. nurses have a high proportion of bank staff). Replacing experienced leavers with newly qualified joiners does not plug the workforce deficits alone—it is critical to also implement strategies to retain experienced staff. Consideration also needs to be given to the speed at which healthcare professionals are trained. The F2 Career Destinations Survey for doctors^33^ shows a rapid decrease in recent years of the proportion of doctors who, 2 years after graduating, continue directly onto the next stage of training. These doctors are not necessarily leaving medicine but are slowing down their progression, either to support personal or professional development,^34^ and/or to manage stress, regain control of their life and work.^35^ Ensuring evidence-based support for staff throughout their training and practice is essential to reduce this attrition.

All four professions experience poor levels of workplace well-being, according to all of the metrics presented in this paper. A notable finding is the difference in sickness absence rates between doctors and the other professions (over a 3-fold difference), a pattern that continues for rates of presenteeism. It is unclear why this is. It may be explained in part by gender socialization theory and gender traits: that it is more acceptable for women to report being stressed than men, and therefore the female dominated professions having higher rates.^36^ This does not explain why the rates are similar in paramedics though who are a more gender-balanced profession. It is more likely a complex interplay of the bio-psycho-social-cultural factors that interact with gender and these professions, for example those with lower income and status being at greater risk of poor mental health. The barriers to taking time off sick may be greater for doctors, including that it may be harder for them to report poor well-being either culturally and/or practically, as they are less likely to be registered with and/or consult with their own GP.^20^^,^^37^ The stigma of mental ill-health and impact on colleagues has been reported by doctors, nurses, midwives and paramedics.^38–40^

Media reports and now published research on experiences of staff during COVID-19 tell us that NHS staff have long been experiencing a mental health crisis, but that has been made significantly worse by the COVID-19 pandemic.^41–43^ This is not reflected in the NHS Staff Survey findings reported here perhaps as the measures were not sensitive to the impact of COVID on the mental health of staff, or because they were collected too early in the pandemic. Increasing support for NHS staff well-being is thus vital. Our current research study Care under Pressure 2 (nurses, midwives and paramedics) will complete summer 2022, and the next steps are to ensure a pathway to impact by embed this research into practice by testing and refining this knowledge and optimizing its implementation in the NHS. To do this we aim to create resources to augment the NHS Health and Wellbeing Framework (HWF).^44^ This Framework was first launched in 2018 by NHS England and Improvement and NHS Employers and provides an interactive toolkit that makes the case for staff health and well-being, sets out clear actionable steps and includes guidance on how organizations can plan and deliver a staff health and well-being strategy. This framework takes a ‘systems and multi layered’ approach to health and well-being (from prevention to treatment, and individual and organizational strategies). Although an excellent resource, currently the NHS HWF has a generic NHS workforce focus (not specifically for doctors, nurses, midwives and paramedics), and our ongoing planned work (through new studies Care Under Pressure 3 and 4) aim to add resources to this framework and optimize their use and implementation in practice.

Through completing this critical review, we have learned that this type of comparative work is not as straightforward as it might seem, that some key data are not available, or need transforming to be comparable for example, but it can generate significant insights, and has significant potential for impact. The findings may help NHS managers, policy makers, leaders, etc. to see where improvement strategies from one profession/setting might be transferrable to another profession/setting, and can also help with targeting/prioritizing the implementation of different initiatives given finite resources (time/money).

This review is limited by the data available, which in some cases is either a few years old and/or has limited comparability across professions. There are important features of work or of the workforce that we do not have reliable data about and therefore could not include: in particular the primary care workforce, which is sizeable, and the settings in which staff work. In addition, sometimes the data do not reflect the true picture on the ground, for example sometimes posts are not advertised because it is not felt they could be filled and workarounds are made to cover service needs, therefore masking the true vacancy rate.

This review presents novel inter-professional comparative work, enabling healthcare leaders, managers and other stakeholders to consider—and develop strategies to mitigate—the potential impact of these distinct demographic and service architecture profiles on well-being of the workforce. Healthcare relies on interdisciplinary working, and attempts to improve workforce well-being require multilevel systems approaches, from prevention to treatment, that take into account similarities and differences across professions. The development of more harmonized inter-professional national databases, could in itself be a resource to monitor and improve healthcare staff well-being.

## Data Availability

The data underlying this article are available in the article and in its online supplementary material.

## Footnotes

1 Care Under Pressure 2: Caring for the Carers a realist review of interventions to minimize the incidence of mental ill-health in nurses, midwives and paramedics National Institute for Health Research Award ID: NIHR129528 https://fundingawards.nihr.ac.uk/award/NIHR129528

2 Care Under Pressure 2: Caring for the Carers a realist review of interventions to minimize the incidence of mental ill-health in nurses, midwives and paramedics National Institute for Health Research Award ID: NIHR129528 https://fundingawards.nihr.ac.uk/award/NIHR129528
